# Complete genome sequences of *Sellimonas intestinalis* JCM 30749^T^, *Sellimonas caecigallum* JCM 35759^T^, and *Sellimonas catena* JCM 35622^T^ and JCM 35623

**DOI:** 10.1128/mra.01282-24

**Published:** 2025-02-25

**Authors:** Kazuyoshi Koike, Kazutoshi Murotomi, Mayu Hamajima, Dieter M. Tourlousse, Atsushi Hisatomi, Mitsuo Sakamoto, Yuji Sekiguchi

**Affiliations:** 1 Biomedical Research Institute, National Institute of Advanced Industrial Science and Technology (AIST), Tsukuba, Ibaraki, Japan; 2 Microbe Division/Japan Collection of Microorganisms, RIKEN BioResource Research Center, Tsukuba, Ibaraki, Japan; Rochester Institute of Technology, Rochester, New York, USA

**Keywords:** *Sellimonas*, complete genome sequence

## Abstract

We obtained complete genome sequences of *Sellimonas intestinalis* JCM 30749^T^, “*Sellimonas caecigallum*” JCM 35759^T^, *Sellimonas catena* JCM 35622^T^, and *Sellimonas catena* JCM 35623. All four genomes consist of a single circular chromosome, with lengths of 2,758,996 to 3,825,976 base pairs and G + C contents of 45.12% to 45.59%.

## ANNOUNCEMENT

The genus *Sellimonas* is a recently cultured, gram-positive bacterium within the family *Lachnospiraceae*, commonly found in the human gut, and is of increasing interest because of its potential impact on human health (1). After reclassification of *S. monacensis* (2), the genus currently contains two validly described species, namely, *S. intestinalis* and *S. catena* (1, 3), as well as two additional effectively published taxa, namely, “*Candidatus* Sellimonas avistercoris” and “*S. caecigallum*” (4, 5). The role of *Sellimonas* in health is complex, with studies suggesting both harmful (e.g., depressive symptoms [6]) and protective (e.g., type 2 diabetes mellitus risk (7) and posttraumatic stress disorder risk [8]) associations. It may serve as a biomarker for gut homeostasis (9) and dysbiosis (10, 11), highlighting its dual influence on health. In this study, we generated complete genome sequences of the type strains of *S. intestinalis* (strain JCM 30749^T^), “*S. caecigallum*” (strain JCM 35759^T^), and *S. catena* (strain JCM 35622^T^), as well as *S. catena* JCM 35623. These strains were previously isolated from human feces (JCM 30749^T^, JCM 35622^T^, and JCM 35623) or chicken cecum (JCM 35759^T^).

Cells were obtained from the Japan Collection of Microorganisms (JCM) and cultured anaerobically in the GAM medium at 37℃ under an atmosphere of N_2_:CO_2_ (80:20, vol/vol). Genomic DNA was extracted from overnight cultures using the Nanobind CBB kit RT (PacBio, Melon Park, CA, USA), following the manufacturer’s protocol, and integrity (peak size of >20 kbp) verified using an Agilent gDNA ScreenTape System with a 4200 TapeStation device (Agilent, Santa Clara, CA, USA). Sequencing libraries were prepared using ONT’s Native Barcoding Kit 24 (SQK-NBD114.24; Oxford Nanopore Technologies, Oxford, UK), without fragmentation and following the manufacturer’s protocol, and sequenced on an R10.4.1 flow cell with a GridION device (Oxford Nanopore Technologies). For all bioinformatics analyses, default parameters were used, unless indicated below. Basecalling was performed using Dorado v0.6.2 with model dna_r10.4.1_e8.2_400bps_sup@v4.3.0 (https://github.com/nanoporetech/dorado). Concatenated reads were identified and split using Duplex Tools v0.3.1 (https://github.com/nanoporetech/duplex-tools). Read data were subsequently demultiplexed using the guppy_barcoder command in Guppy v6.5.7 (Oxford Nanopore Technologies) with concurrent trimming of barcodes (options -enable_trim_barcodes -barcode_kits SQK-NBD114-24). Reads with an average quality of <12 and length of <1,000 bases were discarded using NanoFilt v2.8.0 (12). This yielded 110,509–302,965 reads (793,628,272 to 1,823,353,810 total bases, with average lengths of 3,673–8,149 bases, Table 1). Genome assemblies were generated using Flye v2.9.1 (13), resulting in a single circular contig (as indicated by Flye) for all strains. The assemblies were then directly polished with Medaka v1.7.3 (https://github.com/nanoporetech/medaka) using model r1041_e82_400bps_sup_v4.0.0. Completeness (98.25%–99.42%) and contamination (0.68%–1.17%) were evaluated with the CheckM v1.1.3 lineage_wf (14) (Table 1). Annotation was performed using the NCBI Prokaryotic Genome Annotation Pipeline v6.8 (15).

**TABLE 1:** Summary of the genome sequences generated in this study^*a*^

Species	*Sellimonas intestinalis*	*Sellimonas caecigallum*	*Sellimonas catena*	*Sellimonas catena*
Strain	JCM 30749^T^	JCM 35759^T^	JCM 35622^T^	JCM 35623
Accession no.	CP173694	CP173660	CP173695	CP173661
Circular genome	Yes	Yes	Yes	Yes
No. of contigs	1	1	1	1
Genome size (bp)	3,228,050	2,758,996	3,825,976	3,801,783
GC content (%)	45.37	45.12	45.49	45.59
No. of reads	110,509	216,034	140,072	302,965
N50 of reads (bases)	15,322	5,607	13,103	9,245
Coverage (×)	279	288	286	480
Completeness (%)	98.83	99.42	98.25	98.25
Contamination (%)	0.88	0.68	1.17	1.17
Genes (total)	3,138	2,674	3,793	3,766
CDSs (total)	3,058	2,594	3,713	3,683
Genes (coding)	3,008	2,552	3,600	3,578
CDSs (with protein)	3,008	2,552	3,600	3,578
Genes (RNA)	80	80	80	83
rRNAs (5S, 16S, 23S)	5, 5, 5	5, 5, 5	5, 5, 5	5, 5, 5
Complete rRNAs (5S, 16S, 23S)	5, 5, 5	5, 5, 5	5, 5, 5	5, 5, 5
tRNAs	61	61	61	64
ncRNAs	4	4	4	4

^*a*^ CDS: coding sequence.

The genomes of all four strains consist of single circular chromosome (2,758,996–3,825,976 bp with 45.12–45.59% G + C contents, Table 1). The genomes were annotated to contain 2,674–3,793 protein-coding sequences (Table 1). We expect that these complete genomes of *Sellimonas* species may provide a more reliable basis for exploring their roles in the human gastrointestinal tract.

## Data Availability

All genome sequences have been deposited in DDBJ/EMBL/GenBank under accession numbers CP173694, CP173660, CP173695, and CP173661 for strains JCM 30749^T^, JCM 35759^T^, JCM 35622^T^, and JCM 35623, respectively. Sequencing reads are available in NCBI’s Sequence Read Archive under accession numbers SRR31305267, SRR31305269, SRR31305306, and SRR31305307.
